# Delirium in Adults With COVID-19–Related Acute Respiratory Distress Syndrome: Comparison With Other Etiologies

**DOI:** 10.1212/WNL.0000000000201162

**Published:** 2022-11-15

**Authors:** Raphael Bernard-Valnet, Eva Favre, Adriano Bernini, Mauro Oddo, Jean-Daniel Chiche, Renaud A. Du Pasquier, Andrea O. Rossetti, on behalf of the CORO-NEURO-ICU Study Group

**Affiliations:** From the Neurology Service (R.B.-V., R.A.D.P., A.O.R.), Department of Clinical Neurosciences; Department of Intensive Care Medicine (E.F., J.-D.C.); Neuroscience Critical Care Research Group (A.B.), Department of Intensive Care Medicine; and Medical Direction (M.O.), Lausanne University Hospital (Centre Hospitalier Universitaire Vaudois) and University of Lausanne, Switzerland.

## Abstract

**Background and Objectives:**

Neurologic complications have been associated with COVID-19, including delirium. Such complications have been reported to be frequent among intensive care unit (ICU)-admitted patients. We hypothesized that the rate of neurologic complications would be higher in COVID-19 associated acute respiratory distress syndrome (ARDS) than those who develop ARDS from a different cause.

**Methods:**

We conducted a retrospective cohort study in the adult ICU of Lausanne University Hospital, including all consecutive patients fulfilling the Berlin criteria for ARDS hospitalized between December 2017 and June 2021, stratifying exposure between COVID-19 or not. The primary outcome was delirium onset during ICU stay, defined by the confusion assessment method (CAM-ICU). Exploratory outcomes included development of neurologic complications of the central nervous system (stroke, hemorrhage, and vasculitis), critical illness weakness, and 30- and 180-day all-cause mortality.

**Results:**

Three hundred eleven patients were included in the study (253 with COVID-19 and 58 with other causes) and CAM-ICU could be assessed in 231 (74.3% in COVID-19 vs 74.1% in non-COVID-19). The proportion of patients developing delirium was similar in patients with COVID-19 and controls in univariate comparison (69.1% vs 60.5%, *p* = 0.246). Yet, patients with COVID-19 had a higher body mass index, lower ICU severity, longer mechanical ventilation, and higher sedation doses (propofol and dexmedetomidine). After adjusting for these factors in a multivariable analysis, the risk of delirium remained comparable across groups (adjusted OR [95% CI]: 0.86 [0.35–2.1]). Similarly, COVID-19–related ARDS had no effect on all-cause mortality at 30 days (adjusted OR: 0.87 [0.39–1.92]) and 180 days (adjusted OR: 0.67 [0.33–1.35]). Finally, neurologic complications affecting the CNS (adjusted OR: 1.15 [0.25–5.29]) and critical illness weakness (adjusted OR: 2.99 [0.97–9.1]) were not higher in the COVID-19 group.

**Discussion:**

Compared with other etiologies, patients with COVID-19 did not have higher incidence of delirium and other neurologic complications, after accounting for underlying disease severity in patients with ARDS. Management of COVID-19–associated ARDS needed longer invasive ventilation and higher sedation, which could explain higher rates of delirium in uncontrolled studies.

Coronavirus disease 2019 (COVID-19) caused by severe acute respiratory syndrome coronavirus 2 (SARS-CoV-2) manifests primarily as respiratory symptoms. It had been estimated that up to 17% of hospitalized patients during the first epidemic waves required invasive ventilation, mostly due to development of an acute respiratory distress syndrome (ARDS).^1^ Histologic examinations of autopsy studies revealed that SARS-CoV-2 causes diffuse alveolar damage with severe endothelial injury and capillary microthrombi.^2^ In addition to pulmonary involvement, it has been suggested that SARS-CoV-2 triggers a hyperinflammatory and prothrombotic state that could induce multiorgan involvement.^3^

Neurologic complications in patients with COVID-19 have been reported since the beginning of the pandemic and encompass ICU-related conditions such as neuromyopathy or delirium (also termed in several publications as encephalopathy),^4^ thromboembolic manifestations (ischemic stroke),^5^ or parainfectious complications (such as encephalitis and Guillain-Barré syndrome).^6,7^ In this context, delirium is the most frequently reported neurologic complication, with a frequency of up to 80% of patients with COVID-19 admitted to the intensive care unit (ICU),^8-10^ which represents a high proportion compared with other ICU-related conditions.^11,12^ Delirium is associated with mid- and long-term neurocognitive dysfunction^13,14^ that may account for some of the deficits observed after COVID-19. Although it seems that SARS-CoV-2 does not directly infect the CNS,^15^ some studies suggest that inflammation, hypoxia,^16^ and microvascular damage may affect the blood-brain interface and induce brain dysfunction.^17,18^ In addition to biological mechanisms, some changes in the ICU standard of care (i.e., pain management, sedation/analgesia choice, delirium prevention, early mobilization, and family engagement) in the context of bed shortage could affect the delirium rate.^19,20^ Yet, this increase could also be influenced by the dramatic increase of the number of patients with severe respiratory failure requiring special management during the COVID-19 pandemic, including longer invasive ventilation support and high doses of continuous sedative/analgesic drugs infusions.^21^

To our knowledge, very little attention has been directed to compare the risk of neurologic complications between patients with COVID-19 ARDS compared with other etiologies. Our study aimed to investigate whether delirium and other neurological complications are more frequent in ICU patients developing ARDS related to COVID-19 compared with ARDS from other etiologies, when taking into account disease severity.

## Methods

### Design, Setting, and Participants

We conducted a retrospective cohort study at the Lausanne University Hospital (Centre Hospitalier Universitaire Vaudois [CHUV]), considering COVID-19 as exposure and occurrence of delirium as outcome. All consecutive adults (aged ≥18 years) admitted at the CHUV ICU for more than 24 hours for severe respiratory distress between December 2017 and June 2021 were screened for ARDS. All patients fulfilling the Berlin ARDS criteria^22^ were included. Patients who did not consent to clinical research, were unable to consent, or had had severe prior cognitive impairment (as mentioned in their charts) were excluded.

Data related to the ICU stay were extracted directly from patients' electronic medical records (MetaVision, iMDSoft, Tel Aviv, Israel). Data collected included demographics (age and sex), weight, body mass index (BMI), Simplified Acute Physiology Score II (SAPS II)^23^ on admission, comorbidities at admission (chronic obstructive pulmonary disease [COPD], stroke [or TIA], mild cognitive impairment, heart failure, chronic kidney disease, hypertension, atrial fibrillation, active smoking, diabetes, and coronary disease), PaO_2_/FiO_2_ ratio on admission, worst PaO_2_/FiO_2_ ratio, severity of ARDS (according to the Berlin ARDS criteria^22^), confusion assessment method (CAM-ICU)^24^ (which was only routinely assessed during the ICU stay), total dose (including boluses and continuous infusions) of fentanyl, propofol, midazolam, dexmedetomidine, clonidine, quetiapine, cisatracurium, or haloperidol, coma duration (defined as the number of days with a Richmond Agitation–Sedation Score [RASS] at −5 or −4^25^), steroid use, duration of mechanical ventilation, and neurologic complications. We also retrieved C-reactive protein (CRP) and d-dimer levels as biomarkers of inflammation and thrombosis. Data on survival were extracted from the Swiss federal death registry, allowing to consider death after discharge of our hospital.

### Standard Protocol, Approvals, Registrations, and Patient Consents

This study has been approved by the local ethic committee (CER-VD) in the frame of the CORO-NEURO study (authorization no. 2020-01123). We obtained a consent waiver for deceased patients.

### Management of Analgesia and Sedation

These parameters were standardized according to a local nurse-led protocol in line with current recommendations.^26^ Prescribed drug doses were tailored to the level of sedation required and defined by the RASS. Analgesia was provided using continuous infusion of fentanyl (1–1.5 µg/kg/h). Preemptive boluses of analgesic drugs could be administered before painful procedures. Patients were sedated with propofol (2–4 mg/kg/h) as a first-line treatment; midazolam (0.05–0.15 mg/kg/h) was administrated as second line. When clinically required, a neuromuscular blockade agent (cisatracurium) was administrated in addition to analgesia and sedation.

### Care Organization During COVID-19

To cope with the influx of intensive care patients during the pandemic, health care professionals usually working in other acute care units were requisitioned. They were less familiar with the recommended practices of analgesia and sedation in ICUs but were coached by the ICU staff. Family/friend visitations were strictly prohibited between March 2020 and June 2020 except for end-of-life situations. After June 2020, visitations were restricted to 1 relative, 1 hour per day.

### Outcome Variables

The primary outcome was delirium, assessed through the CAM-ICU routinely performed by nurses twice a day in patients with RASS ≥ −2. Patients were considered delirious if they had at least 1 positive CAM-ICU, as in previous studies.^8,27,28^ We also analyzed delirium length by retrieving the number of days with at least 1 positive CAM-ICU. Yet, these data were censored when the patients were discharged from the ICU. Patients were considered still delirious at discharge if they had at least 1 positive CAM-ICU in the last 48 hours before discharge without 2 consecutive negative assessments.

Patients for whom a CAM-ICU assessment could not be performed were excluded from the analysis of the primary outcome (delirium), but included in analyses of exploratory outcomes, that is, survival at day 30 and at 180, occurrence of complications of the CNS (such as stroke and vasculitis), or critical illness weakness during acute hospital stay. CNS and critical illness weakness were assessed clinically (by critical care or neurology specialists) and reported in the patients' charts. However, there was no standardized screening for critical illness weakness.

### Statistical Analysis

We aimed to test the hypothesis that the incidence of delirium and neurologic complication is higher in COVID-19–associated ARDS than in ARDS from other causes. The rate of delirium was recently estimated at 59% in critically ill patients without COVID-19 in our hospital.^27^ Conversely, previous studies^8,9^ available at the initiation of this project reported delirium rates over 80% in patients with COVID-19. We originally estimated a 2-sided α level of 0.05 and power of 80% to detect an absolute increase of 22% in the delirium incidence in patients with COVID-19; assuming an incidence of 60% in the control group and a 4:1 inclusion ratio (COVID-19:non–COVID-19), at least 180 patients including 144 with COVID-19 vs 36 without COVID-19 were to be enrolled.

Continuous variables are reported as median (interquartile range [IQR] 25%–75%) and categorical variables as numbers and percentages. Comparison across groups was performed using χ^2^ tests for categorical variables and the Student *t* test for continuous variables (when equal variance assumption was violated, the Welch *t* test was used). *p* Values reported were 2 sided, and statistical significance was set at *p* = 0.05.

For the analysis of primary and exploratory outcomes, stepwise binomial logistic regressions were performed. We included in the model the independent variables that were found to be significantly different between groups in the univariate analysis and that were considered relevant for their plausible implication on the outcome (we did not include coma length, as this variable was not considered independent from analgosedation and delirium). We also analyzed survival in a time-dependent manner using a Cox model including the same parameters. To analyze variables associated with delirium, we also ran a binomial logistic regression including demographics, SAPS II, duration of mechanical ventilation, tracheostomy, worst PaO_2_/FiO_2_ ratio, highest CRP during ICU stay, and use of steroids, benzodiazepines, or other sedative agents.

Statistical analyses were performed using SPSS 27 software (IBM, Armonk). Prism 9.0 (GraphPad software, San Diego) was used for graphical representations. Our report follows the Strengthening the Reporting of Observational Studies in Epidemiology guidelines.

### Data Availability

Anonymized data would be made available on reasonable request from qualified and noncommercial entities.

## Results

**Figure 1:**
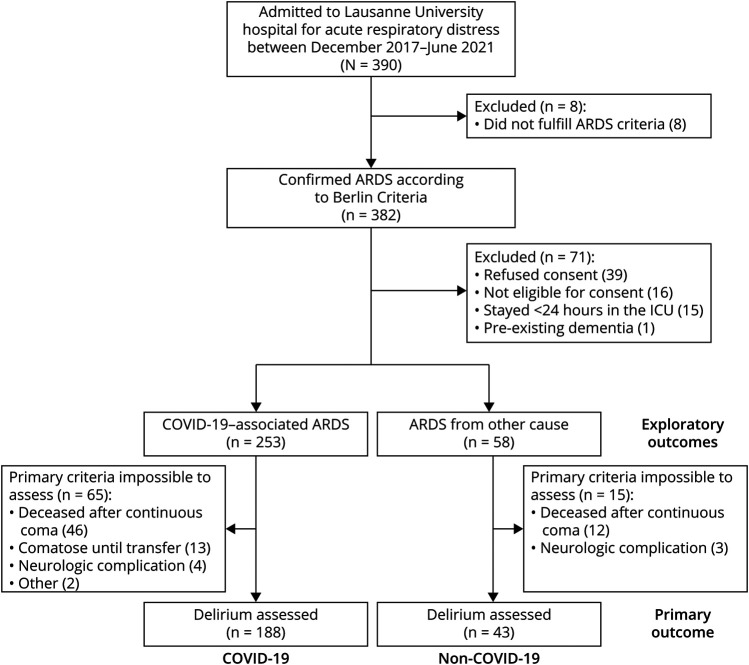
Study Flowchart ARDS = acute respiratory distress syndrome; ICU = intensive care unit.

Three hundred ninety patients were admitted to the ICU at CHUV between December 1, 2017, and May 31, 2021, for severe acute respiratory distress. Of these, 382 fulfilled the Berlin ARDS criteria, and 311 were included for analysis (Figure 1).

**Figure 2:**
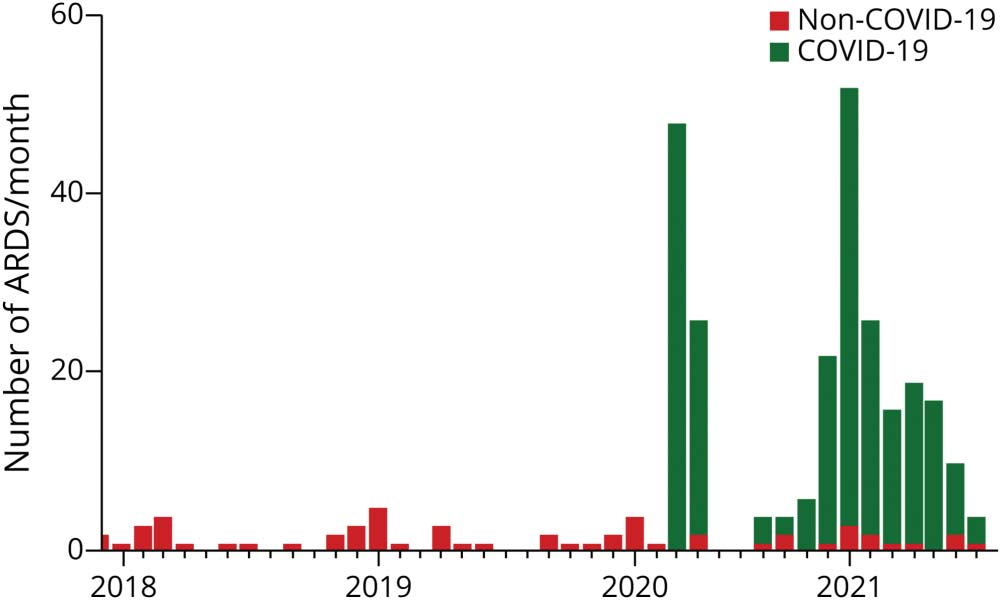
Monthly Incidence of ARDS Number of patients admitted for acute respiratory distress in the ICU per month between December 2017 and May 2021. Patients associated with COVID-19–associated ARDS are depicted in green and ARDS from other cause in red. ARDS = acute respiratory distress syndrome.

In our center, the density of severe ARDS drastically increased during the COVID-19 pandemic, rising from a median incidence of 1 (IQR 0–2) case per month for ARDS from other etiologies to 16 (IQR 4–26) (Figure 2). In patients without COVID-19, pneumonia was the main cause (86.2%).

Baseline and clinical characteristics of the overall cohort (311 patients) are illustrated in Table 1. The median age did not differ between groups (66 vs 66 years, *p* = 0.155). Similarly, except for COPD (20.7 vs 9.1%, *p* = 0.012), the comorbidities at admission were equally distributed among groups, especially mild cognitive impairment (4.7 vs 3.4%, *p* = 0.668) and history of stroke (or TIA) (5.9 vs 3.4, *p* = 0.454).

**Table 1:**
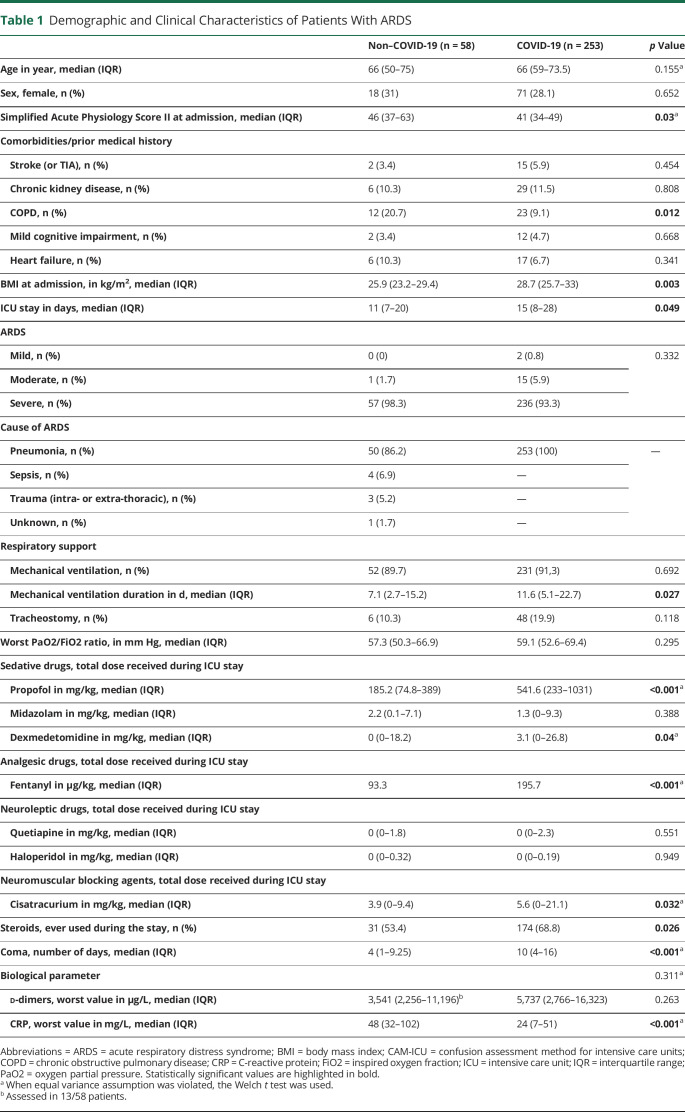
Demographic and Clinical Characteristics of Patients With ARDS

The vast majority of patients in both groups developed a severe ARDS (93.3% vs 98.8%, *p* = 0.332), and there was no differences in worst PaO2/FiO2 ratio (59.1 vs 57.7 mm Hg, *p* = 0.295). However, compared with controls, patients with COVID-19 had a significantly lower SAPSII (46 vs 41, *p* = 0.030) and higher BMI (28.7 vs 25.1, *p* = 0.03). Overall, patients with COVID-19 needed longer mechanical ventilation (11.6 vs 7.1 days, *p* = 0.027), required higher total doses of analgosedation (propofol and fentanyl). The duration in coma (10 vs 4 days, *p* < 0.001) and of ICU stay (15 vs 11 days, *p* = 0.049) were longer in this group (Table 1). Overall use of neuromuscular blocking agents was also higher in the COVID-19 group (5.6 vs 3.9 mg/kg, *p* = 0.032). Neither the proportion of patients receiving benzodiazepine-based sedation (70% vs 72.4%, *p* = 0.712) nor its total dose (Table 1) was different between groups. Patients without COVID-19 had higher CRP levels (24 vs 48 mg/L, *p* < 0.001). Only 13 non-COVID patients had d-dimers assessed during their ICU stay. However, when measured, d-dimers were comparable in the 2 groups.

The primary outcome (delirium) could be assessed in 231 patients, and the proportion of patients with impossibility to assess delirium was similar between the 2 groups (25.7 vs 25.9%, *p* = 0.949). Reasons that prevented delirium evaluation are summarized in Figure 1. Of note, there were no patients with recent (<30 days) acute brain injury on admission (including patients with coma after traumatic brain injury, ischemic/hemorrhagic stroke, cardiac arrest, and status epilepticus) evaluated for the primary outcome in both groups. Clinical characteristics of assessed patients did not differ from the whole cohort (eTable 1, links.lww.com/WNL/C270). The incidence of delirium was higher in patients with ARDS related to COVID-19 (69.1%) than from other causes (60.5%), but it was not statistically significant (unadjusted OR (95% CI): 1,47 (0.74–2.91), *p* = 0.246), even after adjusting for SAPSII, BMI, duration of mechanical ventilation, doses of propofol and dexmedetomidine, and steroid use (adjusted OR [95% CI] 0.86 [0.35–2.1], *p* = 0.747) (Table 2).

**Table 2:**
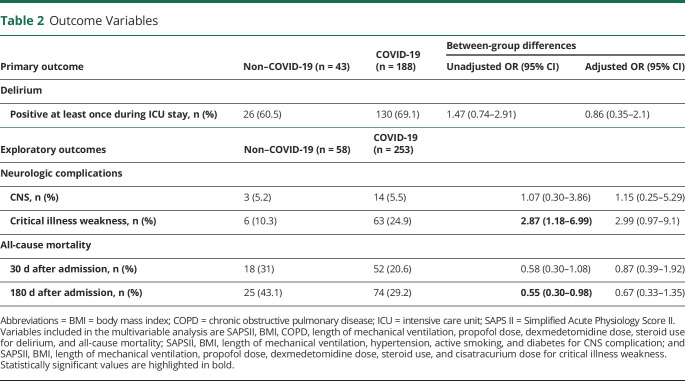
Outcome Variables

The overall duration of delirium in the ICU was also similar between groups (median [IQR] 3 (2–5) vs 3 (2–5) days, *p* = 0.396—data not shown). Forty-three (22.9%) patients with COVID-19 and 9 (20.9%) patients without COVID-19 were considered still delirious at discharge from the ICU (*p* = 0.843).

**Figure 3:**
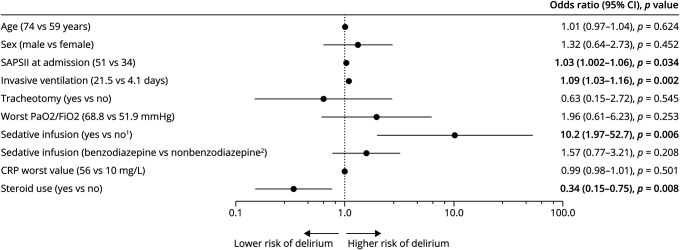
Independent Factors Associated With Delirium In patients assessed for delirium with CAM-ICU (COVID: 188; non-COVID: 43), a binomial logistic regression assessed the effect on delirium of the following parameters: age, sex, SAPS II, length of invasive ventilation, tracheotomy, worst PaO2/FiO2, sedative infusion, benzodiazepine use, worst C-reactive protein (CRP) level, and use of steroids. For all continuous variables (age, SAPSII, length of invasive ventilation, worst PaO2/FiO2, and worst C-reactive protein [CRP]), comparisons shown in parentheses correspond to the 75th vs 25th percentile values for these variables. Statistically significant differences are highlighted in bold. ^1^No infusion of sedative drugs (including propofol, dexmedetomidine, or midazolam). ^2^Use of non-benzodiazepine sedative drugs (propofol and dexmedetomidine) or no sedative drugs. Legend: SAPS II: Simplified Acute Physiology Score II, CRP: C-reactive protein, PaO2: oxygen partial pressure, FiO2: inspired oxygen fraction, CAM = confusion assessment method; ICU = intensive care unit; SAPS II = Simplified Acute Physiology Score II.

We analyzed the factors independently associated with a higher risk of developing delirium assessed by the CAM-ICU in our sample (231 patients). SAPS II, length of invasive ventilation, and use of sedative drugs were associated with a higher risk of developing delirium (Figure 3). Of note, benzodiazepine use (i.e., midazolam) did not lead to a higher delirium rate (OR [95% CI] 1.57 [0.77–3.21], *p* = 0.208), whereas steroids had a protective role (OR [95% CI] 0.34 [0.15–0.75], *p* = 0.008). Given the low number of patients (eTable 1, links.lww.com/WNL/C270) with mild cognitive impairment, we did not assess it as a risk factor for delirium. However, all 9 patients with mild cognitive impairment developed delirium during their ICU stay.

On the whole cohort of 311 patients, complications involving the CNS were relatively uncommon in both groups (Table 2), occurring in 3 controls (5.2%, 2 ischemic strokes and 1 subarachnoid hemorrhage) and 14 patients with COVID-19 (5.5%, 9 ischemic stokes, 2 hemorrhagic strokes, 2 subarachnoid hemorrhages, and 1 CNS vasculitis). The overall risk of CNS complications was not increased in individuals with COVID-19 (unadjusted OR [95% CI] 1.07 [0.30–3.86], adjusted OR [95% CI] 1.15 [0.25–5.29], *p* = 0.857). Risk factors usually associated with cerebrovascular events are depicted in eTable 2, links.lww.com/WNL/C270. Despite no systematic evaluation, critical illness weakness tended to be more frequent in patients with COVID-19 than non–COVID-19 counterparts (unadjusted OR [95% CI] 2.87 [1.18–6.99], adjusted OR [95% CI] 2.99 [0.97–9.1]).

**Figure 4:**
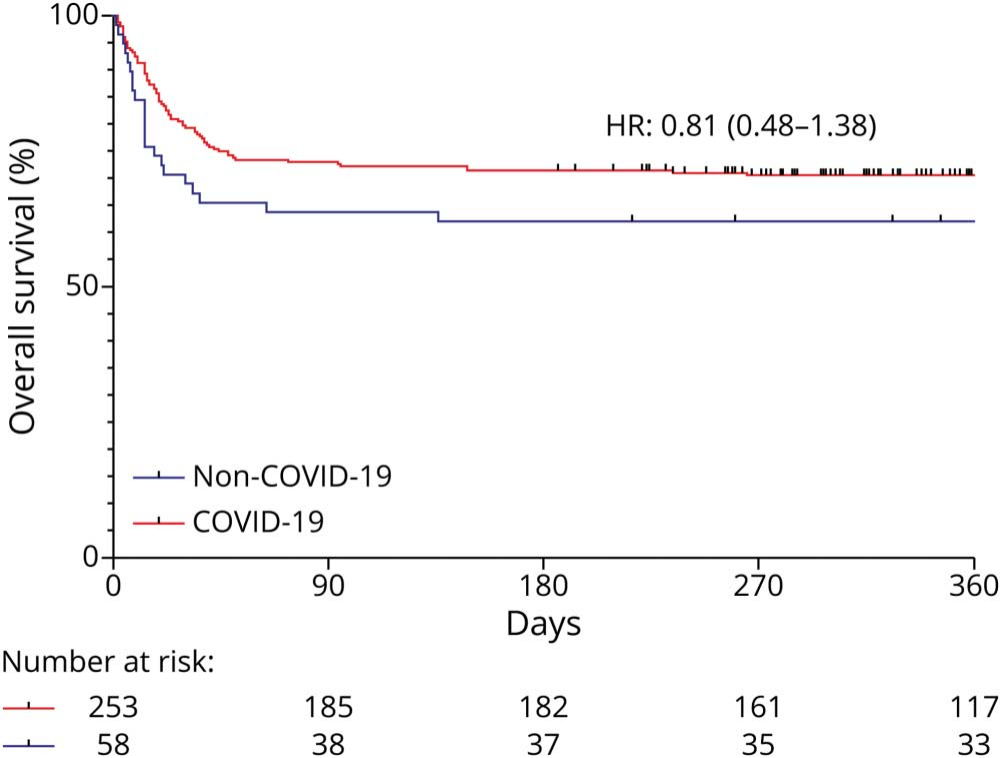
Survival During the First Year After Admission to ICU for ARDS Hazard ratio (HR) provided has been calculated from a cox model adjusted for SAPSII, BMI, length of mechanical ventilation, propofol dose, dexmedetomidine dose, and steroid use. Unadjusted HR: 0.65 (0.4–1.02).

All-cause mortality did not differ between groups at 30 (unadjusted OR [95% CI] 0.58 [0.30–1.08], adjusted OR [95% CI] 0.87 [0.39–1.92]) and 180 days (unadjusted OR [95% CI] 0.55 [0.30–0.98], adjusted OR [95% CI] 0.67 [0.33–1.35]) after admission (Table 2). Similar results were found analyzing survival in a time-dependent manner using a Cox model adjusted for the abovementioned variables (unadjusted hazard ratio: 0.65 (95% CI 0.4–1.02), adjusted hazard ratio: 0.81 (95% CI 0.48–1.38)) (Figure 4).

## Discussion

This study represents an attempt to compare the advent of delirium and neurologic complications between 2 populations of ARDS related or not to COVID-19. Our results suggest that patients with COVID-19 ARDS were not more prone to develop this type of acute brain dysfunction than individuals with ARDS from other etiologies after adjustment for variables known to be associated with delirium. Similarly, they were not at higher risk of developing neurologic complications (either from the CNS or critical illness weakness) or all-cause mortality.

Even if the incidence of delirium was comparable across groups, it is of importance to note the striking difference between the management of ARDS related to COVID-19 and ARDS related to other etiologies substantiating the need for adjustments in the analyses of these 2 groups. Patients with COVID-19 ARDS stayed longer on mechanical ventilation and requested a higher dose of sedative/analgesic drugs. Similarly, patients with COVID-19 were comatose for a longer duration, as previously illustrated.^28^ These parameters have been shown in our study and other cohorts to be independently associated with the development of delirium.^8,28^ Thus, differences in the management of COVID-19 ARDS could explain variations in the delirium incidence, ranging from 55%^28^ to 84%^8^ and reaching 69% in our cohort. It is also possible that these differences could reflect the use of benzodiazepines, which have been shown to predispose for delirium.^28^ Indeed, the 2 studies with a higher level of delirium report higher proportions of benzodiazepine use (86.4%^8^ and 78.4%^9^ against 70% in our cohort and 64% in another one^28^). Yet, in our analysis, benzodiazepine use was not significantly associated with higher delirium. Although the tendency may not have resulted significant in view of the relatively limited sample size, this might also reflect the application of our local analgosedation protocol recommending limitation of benzodiazepine use. However, we were not able ascertain the level of compliance to this protocol during the COVID-19 pandemic. There was no difference between groups for delirium in the univariate analysis, suggesting that other key factors may account for delirium development, such as hypoxia, initial illness severity, or systemic inflammation (given the protective effect of steroids), as outlined in Figure. 4.

SARS-CoV-2 has been initially suggested to directly attack the CNS, especially the brainstem. However, subsequent studies failed to demonstrate any invasive SARS-CoV-2 infection within the CNS.^15,29^ It was also proposed that SARS-CoV-2 affects the brain by indirect mechanisms, such as inflammation, hypoxia,^16^ or development of a prothrombotic state. We previously demonstrated, among other groups,^30^ that patients with COVID-19 with delirium exhibit a higher level of interleukin-8 (IL-8) and chemokine ligand 2 (CCL2) in the CSF correlating with peripheral inflammation.^17^ This was associated with signs of blood-brain barrier dysfunction^17^ and strong glial (astrocytes and microglia) activation. However, these features are not unique to patients with COVID-19: even if the pathophysiology of delirium is still poorly understood, it has been suggested that peripheral inflammation (notably IL-1β) could lead to blood-brain barrier dysfunction and induce a glial reactivity that in turn produces cytokines like IL-8 and CCL2.^31-33^ This neuroinflammation would subsequently trigger a reduction in the cerebral metabolism, thus leading to delirium.^33-35^ These findings are substantiated by our results that do not show a higher rate of delirium in ARDS associated with COVID-19 after adjusting for confounders. Use of steroids was strongly associated with reduced occurence of delirium, further supporting a role for peripheral and CNS inflammation in its development. Indeed, in patients with COVID-19, high-dose steroids have been shown to exert a beneficial effect on encephalopathy.^36^

It has also been proposed that COVID-19 could induce a prothrombotic state and trigger cerebral ischemic and hemorrhagic consequences.^37-39^ With a small number of neurologic complications, we could not demonstrate differences compared with ARDS from other causes. The early use of anticoagulation to prevent arterial and venous complications in the context of prothrombotic state might explain this observation.

Conversely, the rate of critical illness weakness was higher in patients with COVID-19, but it was mainly explained by the longer time on invasive ventilation and the higher use of steroids and neuromuscular blocking agents, which are well-recognized risk factors for this entity.^40,41^ Yet, we have to acknowledge that the incidence of critical illness weakness was low in our cohort.^42,43^ As there was no standardized evaluation, we may hypothesize that only the most severe forms were reported. However, the evaluation and reporting have been similar between the 2 groups.

Regarding mortality, the median SAPS II was higher in patients without COVID-19 (median [IQR] 46 [37–63] than in patients with COVID-19 (median [IQR] 41 [34–49]), resulting in an estimated ICU mortality of 36% and 26%, respectively. This might partially explain the lower survival in patients without COVID-19 that was not significant in multivariable analyses correcting for SAPS II. Nevertheless, our mortality rate seems congruent with other centers.^12,44^

Our study has several limitations. First, its retrospective design and the relatively limited patient sample size may have reduced the sensitivity to detect smaller differences in delirium incidence. Our sample assumption was based on 2 single-center studies reporting a high delirium incidence in this population.^8,9^ Differences between groups could have been missed because of the lack of statistical power. Furthermore, information regarding the preexisting comorbidities was limited. However, major cognitive disorders was an exclusion criteria, and was only assessed retrospectively on patients' charts. Indeed, premorbid frailty including advanced age, dementia, alcohol abuse, and vision/hearing loss are known to play a role in development of delirium.^31^ Similarly, we were not able to assess important factors that could have affected delirium prevention, including early mobilization, physical restraints use, or visit from family/friends.^14,26^ Although this last parameter has been shown to play a role in delirium prevention, family visits were strongly limited for patients with COVID-19 but not for ARDS of the pre-COVID era.^28^ Furthermore, we only had limited information regarding delirium type (hypo- vs hyperactive) and the compliance to prescribed sedation levels. We were also not able to reliably compare delirium duration, for the sake of data quality, we limited our analysis to the ICU stay (as CAM assessments were not routinely performed outside the ICU), and many patients were discharged still delirious. Around 25% of the ARDS cohort patients could not be assessed for delirium (the vast majority died in coma), but the proportion was nearly identical across the 2 groups, strengthening the internal validity of the study. The overall proportion of patients with delirium that seems comparable to the largest study to date^28^ suggests generalizability of our results. Yet, contrary to many other centers, the Lausanne University Hospital was not significantly affected by sedative drugs shortage. This might partially explain some discrepancies in the delirium rate to other studies performed in centers strongly affected by beds and drugs shortages. Finally, patients with COVID-19 included in this analysis were infected with the alpha or delta variant of SARS-CoV-2 but not the highly contagious, but less severe, omicron variant.

To conclude, this controlled study comparing ARDS due to COVID-19 and other causes suggests that the risk of neurologic complications of these patients is similar, including development of delirium. Given the long-term effect of ICU stay and delirium on cognition,^13^ and the massive increase in patients with ARDS over this last 2 years due to the pandemic, COVID-19 neurologic sequelae should be evaluated in large, prospective, long-term assessments.^14,45^

## Glossary

ARDS: acute respiratory distress syndrome
BMI: body mass index
CAM: confusion assessment method
CCL: chemokine ligand
CHUV: Centre Hospitalier Universitaire Vaudois
COPD: chronic obstructive pulmonary disease
COVID-19: coronavirus disease 2019
CRP: C-reactive protein
ICU: intensive care unit
IL: interleukin
IQR: interquartile range
RASS: Richmond Agitation–Sedation Score
SAPS II: Simplified Acute Physiology Score II
SARS-CoV-2: severe acute respiratory syndrome coronavirus 2
